# Diagnosis and management of a giant ovarian cyst in the gravid-puerperium period: a case report

**DOI:** 10.1186/s12884-019-2678-8

**Published:** 2019-12-26

**Authors:** Sibraogo Kiemtoré, Hyacinthe Zamané, Yobi Alexis Sawadogo, Rodrigue Sansan Sib, Evelyne Komboigo, Ali Ouédraogo, Blandine Bonané

**Affiliations:** 1 Training and Research Unit in Health Sciences, University Joseph Ki-Zerbo, 7021, Ouagadougou 03, BP Burkina Faso; 2 Yalgado Ouedraogo teaching hospital, 7022, Ouagadougou 03, BP Burkina Faso; 3 School of Health Sciences, Polytechnic University of Ouahigouya, Ouagadougou 03, BP 36 Burkina Faso

**Keywords:** Giant ovarian cyst, Gravid-puerperium period, Serous cystadenoma

## Abstract

**Background:**

Giant ovarian cyst is very rare in gravid-puerperium period. It is a cause of a maternal-fetal morbidity. We report a case of a giant benign ovarian cyst in gravid-puerperium period which was diagnosed and managed in a hospital of a low-resource country.

**Case presentation:**

Data were collected by historical review, clinical examination, laboratory investigations, imaging examination, and by histopathological study of the excised surgical specimen. It is the case of a 25-year-old woman who was third gravida and third para with unknown pathological history. After she had given birth through vagina, a giant ovarian cyst, unknown during pregnancy, was diagnosed. A left oophorectomy carrying the cyst was performed after laparotomy in Yalgado Ouedraogo University Hospital Center of Ouagadougou (Burkina Faso). The cyst was 42 cm long and weighed 19.7 kg. The histology of the operative specimen revealed serous cystadenoma of the ovary. The postoperative course was uneventful.

**Conclusion:**

This case reports that vaginal delivery is possible with a giant ovarian cyst associated with pregnancy. Surgical management of the cyst can be performed in the postpartum with satisfaction.

## Introduction

Adnexal masses during pregnancy are common. Indeed, 0.2–2% of pregnancies are accompanied by ovarian cysts [1] which are often small and without symptoms. Those cysts are generally discovered incidentally during an ultrasound surveillance of pregnancy or when they become symptomatic. They are often functional and usually resolve spontaneously before the 3rd trimester of pregnancy. Their persistence until the end of pregnancy is in favor of the organic nature of the cyst. Therefore, Adnexal masses measuring more than 5 cm, especially with a solid component in imaging, are most likely to be non-functional [2]. They can rarely become large. A giant ovarian cyst is fluid-filled sac or pocket measuring more than 10 cm developed at the expense of the female gonad [3, 4]. It is very rare in the gravid-puerperium period and is often functional [5]. During pregnancy, this may not be discovered in the African context where ultrasound scans are rarely performed. The presence of an abdominopelvic mass in the postpartum may be a circumstance of discovery. In this work, we report the greatest ovarian cyst ever described in gravid-puerperium period.

## Case presentation

### Sociodemographic characteristics of the patient

The patient was a 25-year-old woman who dropped school early and got married with a single farmer. She lived with her husband in Kongoussi, a rural commune about 100 km from Ouagadougou, the capital city of Burkina Faso. She did not have any income-generating activity. She had her first menses at 16 years old. She was third gravida and third para with three living children.

### Diagnostic approach

For her previous pregnancy, the date of the patient’s last menses was not known and no dating ultrasound was performed. She received prenatal care at the health center of her village, but no paraclinic investigation was carried out during her pregnancy.

**Fig. 1:**
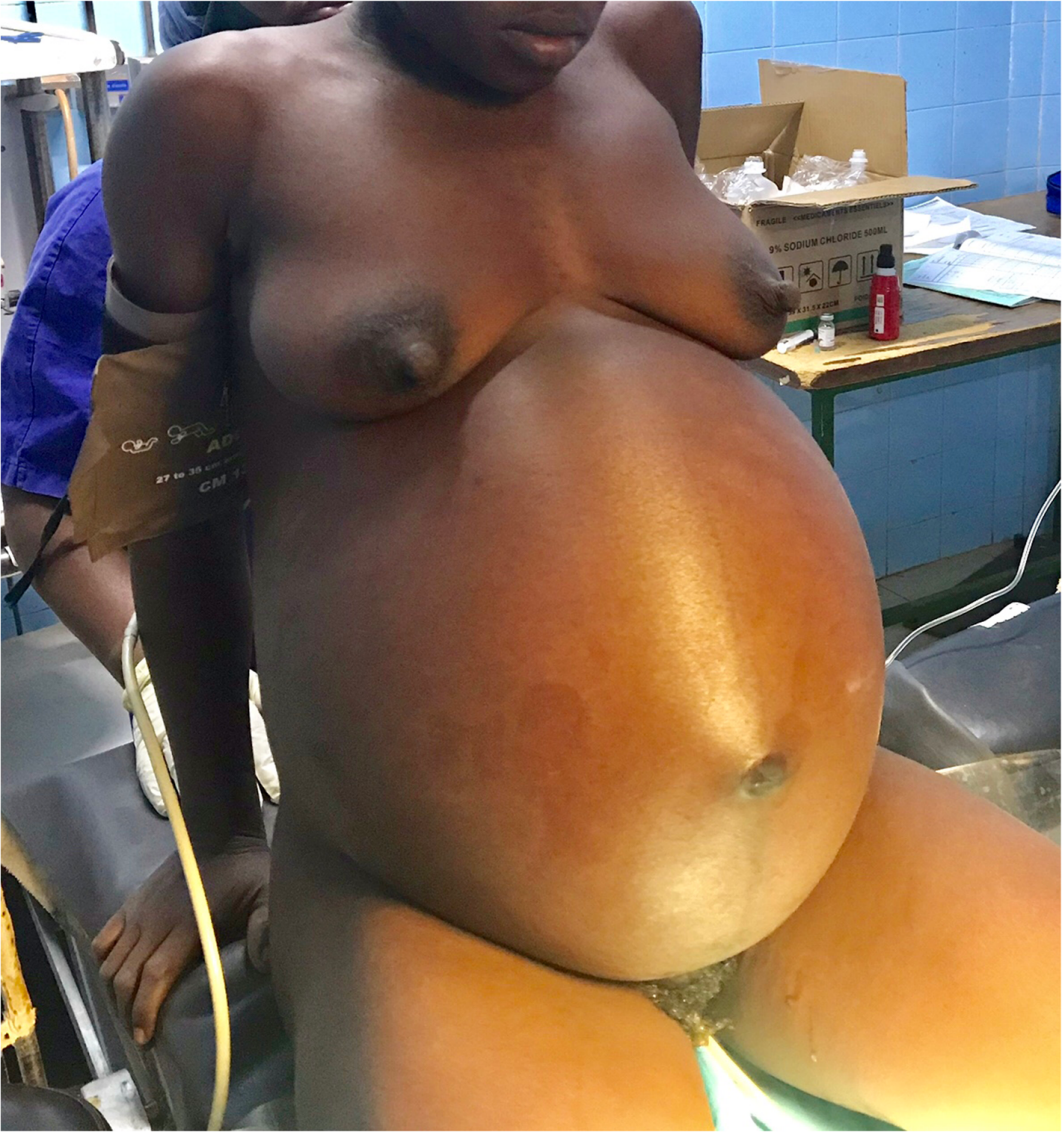
Abdominal distension before removal of abdominal cyst

On November 18th, 2018, the patient gave birth to a baby boy at health center of her village. At birth, the baby had Apgar’s score of 8/10 at the first minute, 10/10 at the 5th minute, and weighed 2780 g. The patient reported that after her last delivery her abdomen had remained large as compared to previous deliveries. She also said to have noticed a gradual increase in the volume of her abdomen in the days following delivery. As she unsuccessfully went through traditional care made of decoction, 10 days after giving birth, she decided to go back to the health center of her village for consultation. From this center, she was referred to the Department of Obstetrics Gynecology at Yalgado Ouedraogo University Hospital Center in Ouagadougou, 27 days after giving birth, i.e. on December 15, 2018. At admission, she was complaining of tension-type abdominal pain and was dyspnetic. She weighed 93 kg and was 167 cm tall with a temperature of 37°2 C. On inspection, the abdomen was largely distended (Fig. 1). As for the palpation, it revealed an enormous abdominopelvic mass with a dull note on percussion. The abdominal perimeter was 126 cm long.

**Fig. 2:**
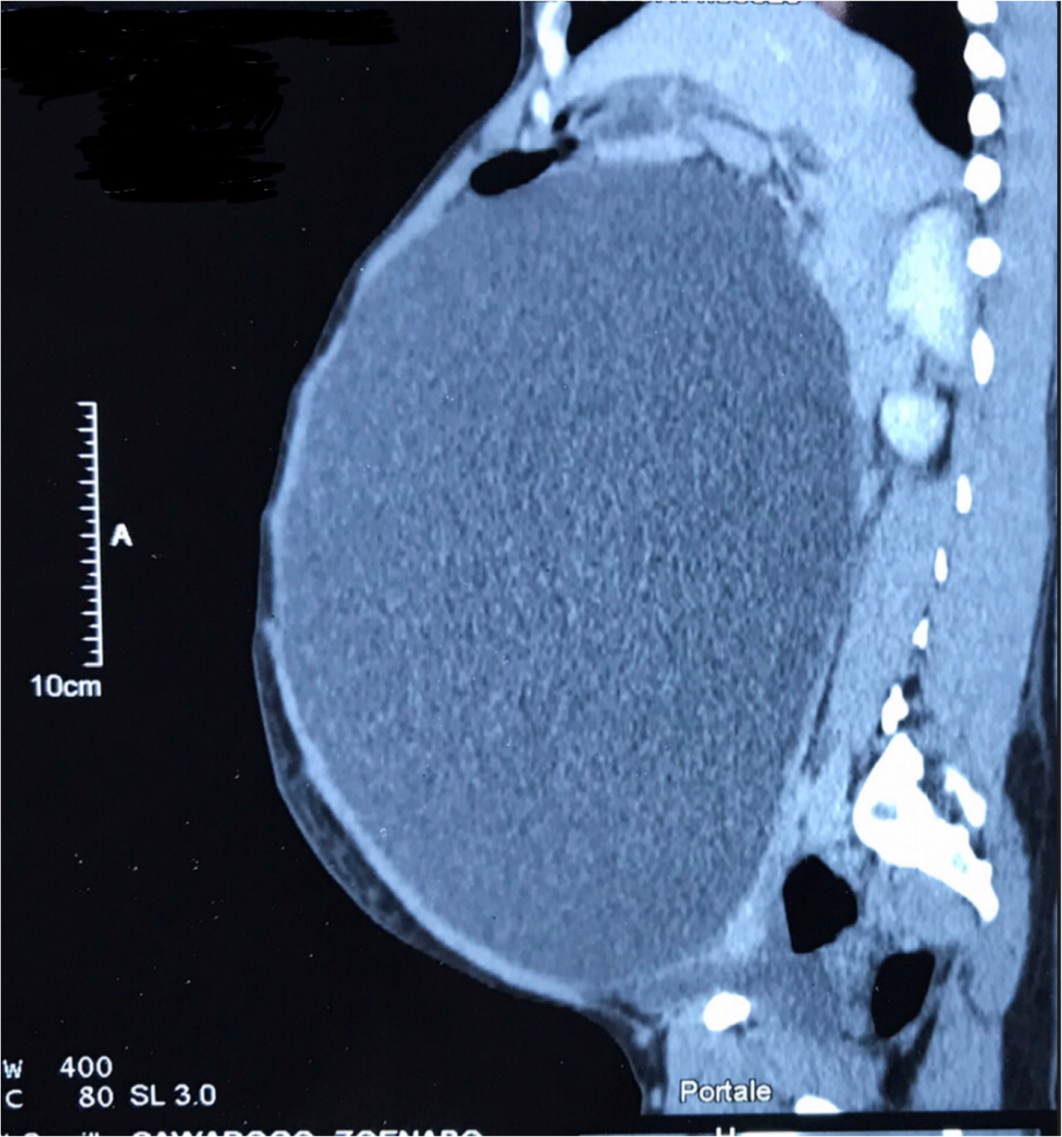
abdominopelvic unilocular cyst by CT scan

An ultrasound found a cystic abdominopelvic image without being able to attach it to an organ. It was then that a Computed tomography (CT) was performed. This CT also showed a fluid mass with no evidence of solid components or septations (Fig. 2). In this regard, the radiologist discussed two diagnoses: a giant ovarian cyst and a huge mesenteric cyst. The ﻿tumor marker CA-125 was normal and the haemoglobin blood level was 14.5 g per deciliter.

### Treatment

**Fig. 3:**
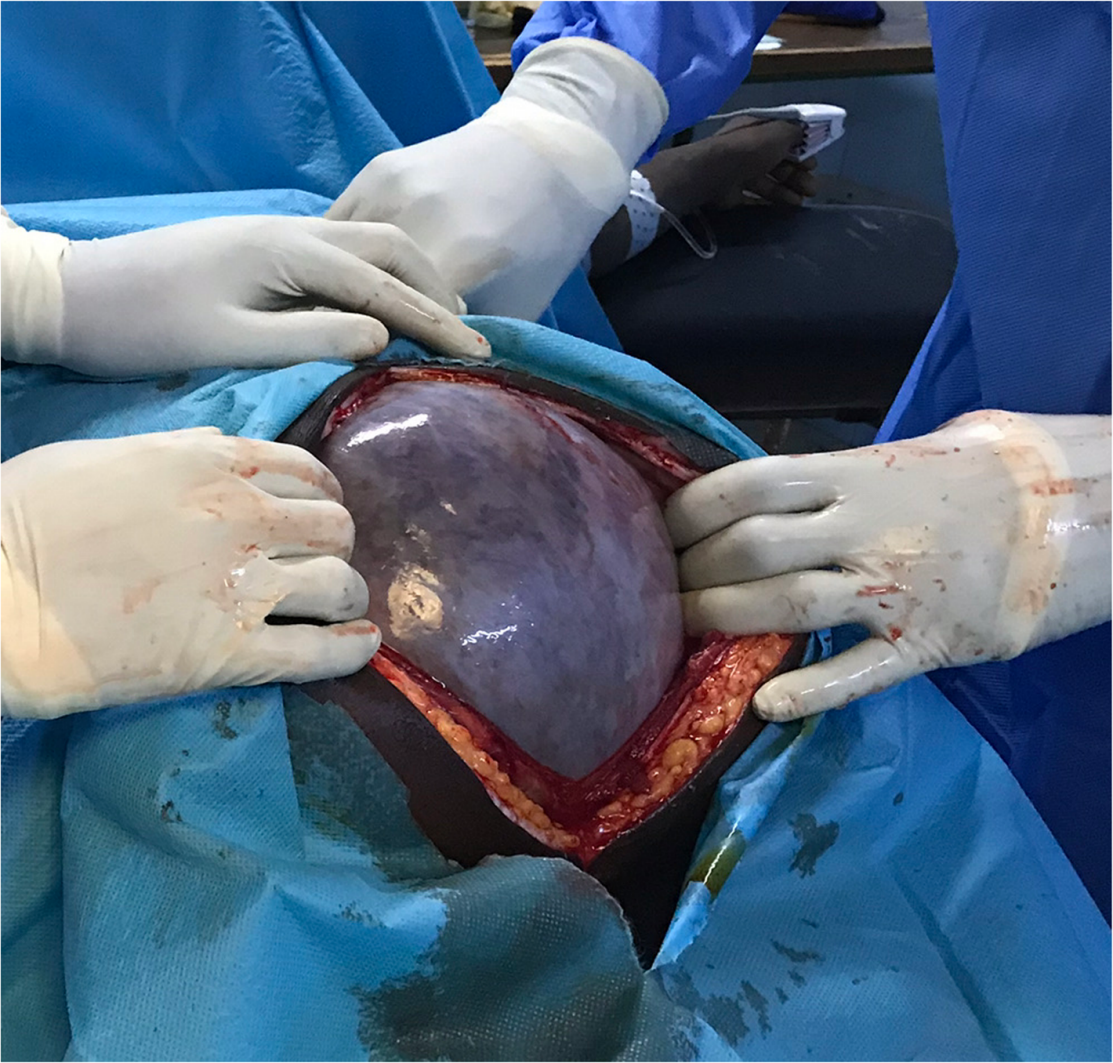
incision from in the lower abdomen up to the umbilicus

**Fig. 4:**
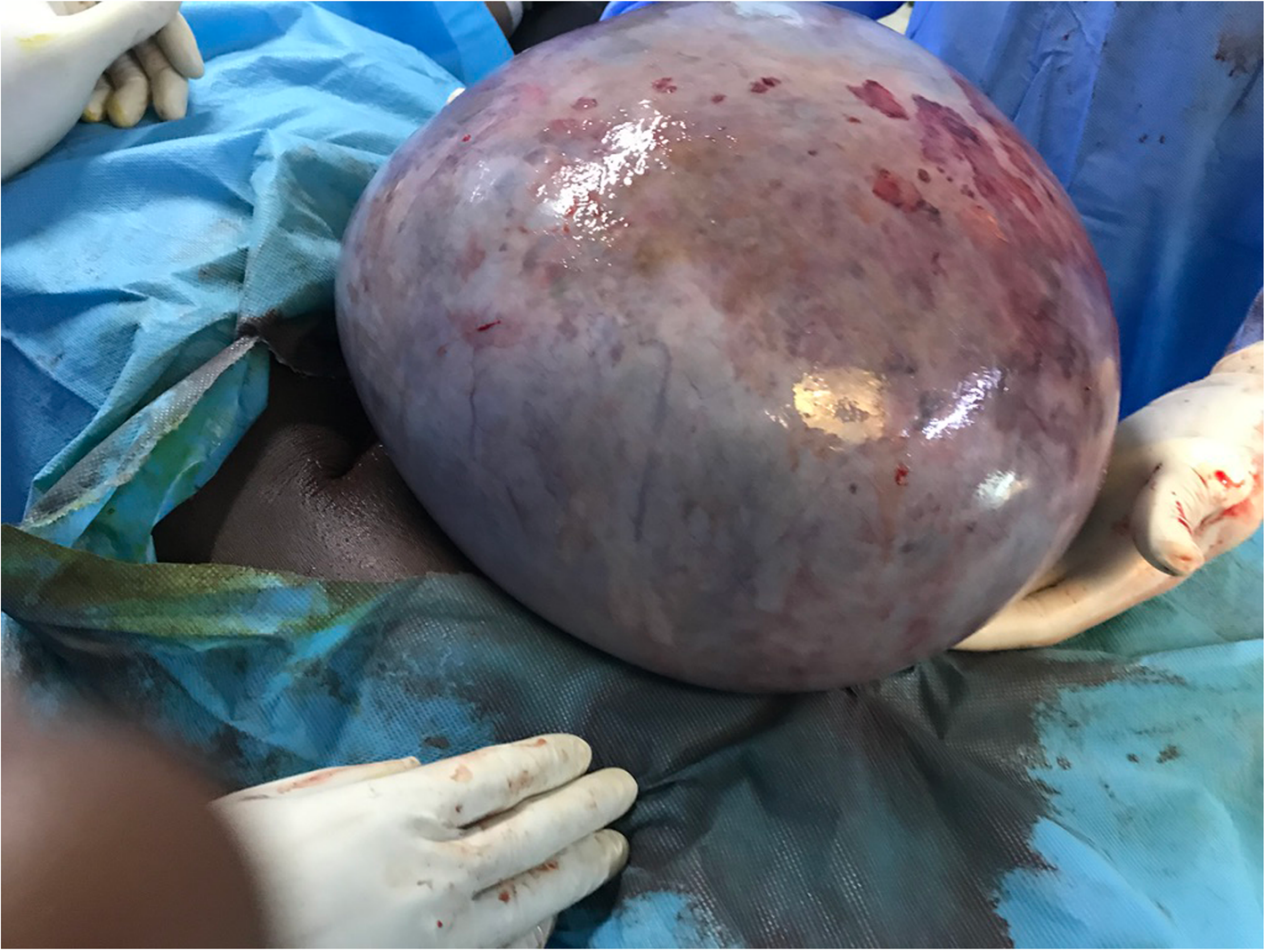
externalization of the giant cyst

**Fig. 5:**
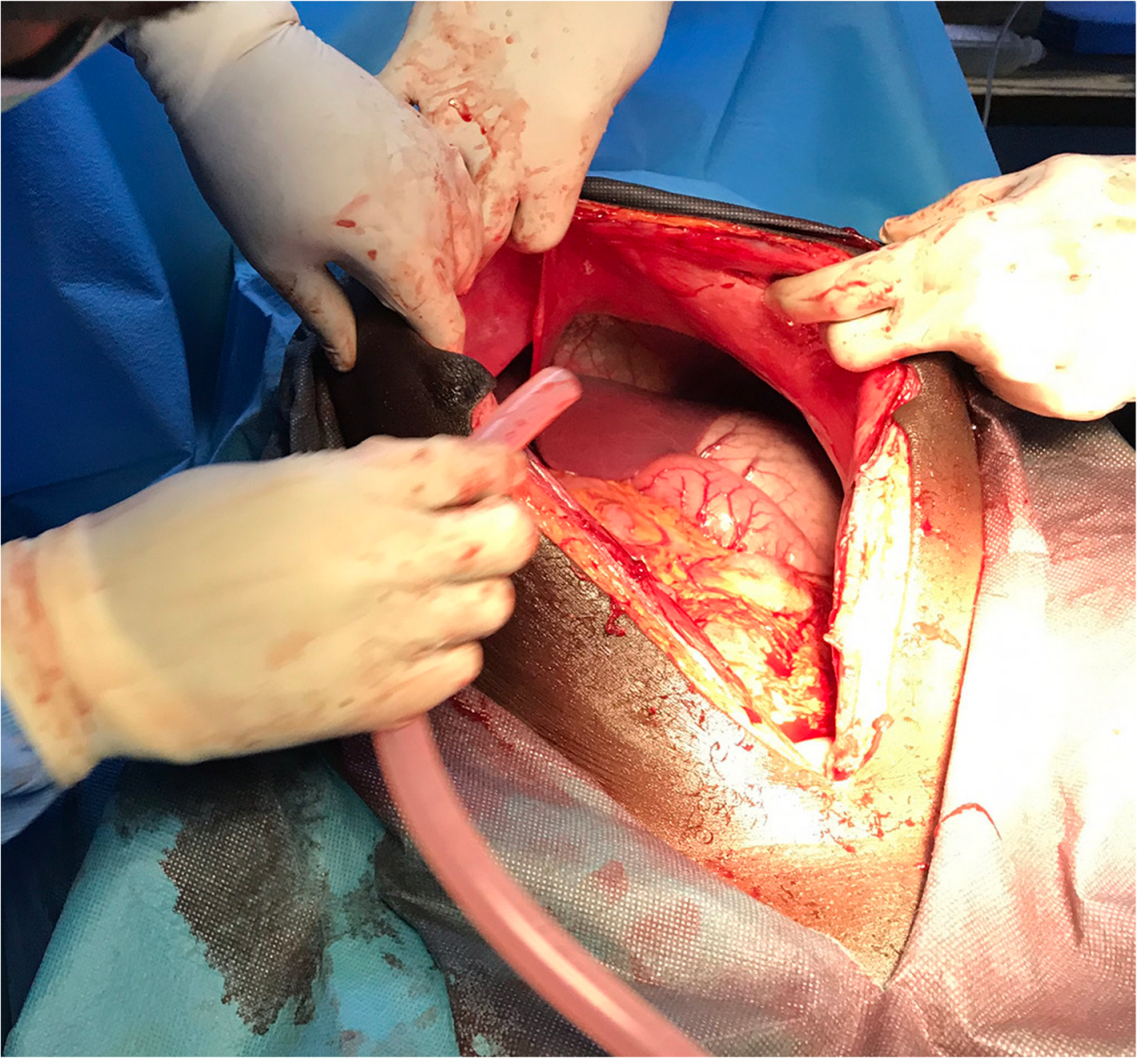
exploration of the entire abdominal cavity; no ascites, no abnormalities of the other intraperitoneal organs found

**Fig. 6:**
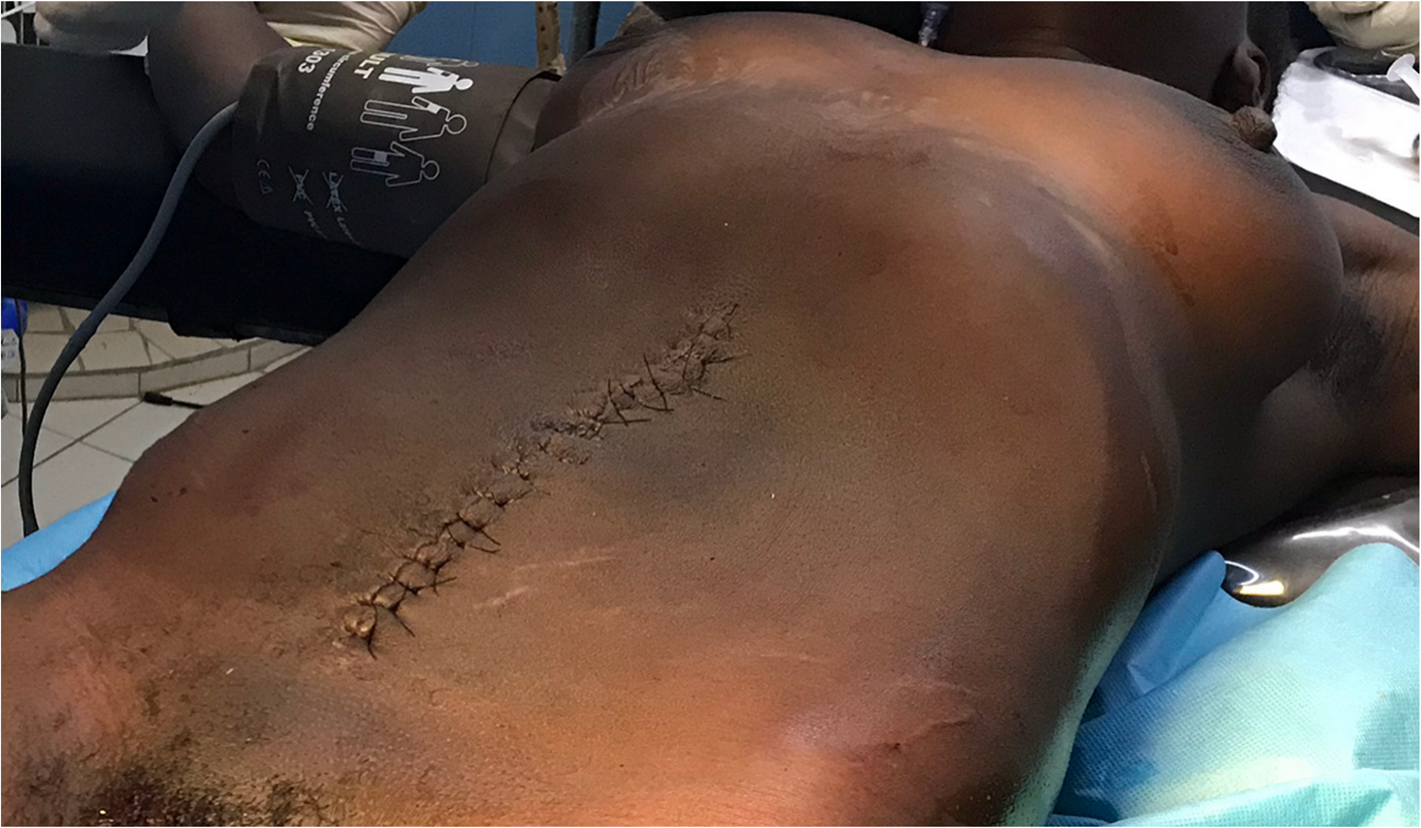
abdomen turned flat immediately after surgery

**Fig. 7:**
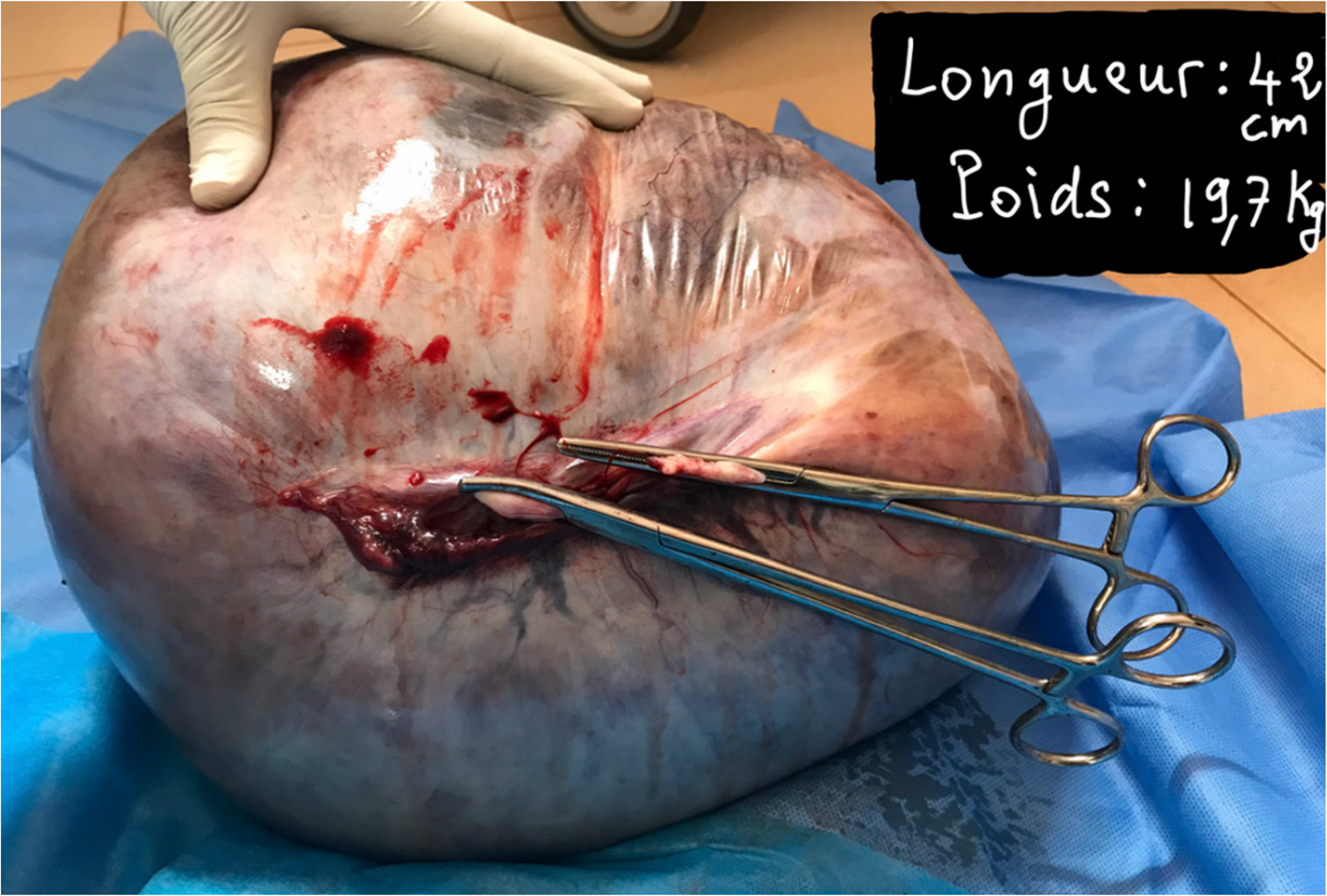
giant ovarian cyst after removal (19,7 kg and 42 in cm in diameter)

We performed a midline laparotomy from the lower abdomen up to the umbilicus (Fig .3) that allowed the externalization of the cyst located in the left ovary (Fig. 4). The uterus and contralateral adnexa were macroscopically normal. We then carried out a left total oophorectomy. The removal of the latter giant ovarian cyst allowed an exploration of the entire abdominal cavity. But we did not find any ascites or abnormalities of the other intraperitoneal organs (Fig. 5). The abdominal wall was closed by some simple interrupted sutures. The fascia was closed with decimal 4 polyglactin sutures and the skin with decimal 3 non-absorbable sutures. The patient’s abdomen turned flat immediately after surgery (Fig. 6). The removed cyst measured 42 cm long-axis and weighed 19.7 kg (Fig. 7). The postoperative period was uneventful and the patient was released from the hospital on the 3rd day after surgery. No complications were observed in the 45 days after surgery. At the histological study, the cyst was benign and was viewed as a serous ovarian cystadenoma.

## Discussion and conclusions

Ovarian cysts occur to 4.9% of pregnant women [5]. Ovarian cysts of the giant-type are extremely rare in the gravid-puerperium period. They represent less than 1‰ of the set of cysts associated with pregnancy [6] and their symptoms are non-specific. Generally, such symptoms as abdomen discomfort, constipation, back or abdominal pain are attributed to regular manifestations of pregnancy. So, these symptoms may be neglected by both the patient and the physician. The following could help discover the cyst before it became large:

- a complication such as torsion. This torsion often occurs when the cyst has between 6 and 8 cm diameter, with almost 60% of instances happening during 10–17 weeks of gestation.

- a systematic ultrasound carried out for the monitoring of the pregnancy. However, the place where our patient resided, no ultrasound was available.

Imaging plays a big role in diagnosis. Compared to ultrasound, Computed tomography (CT) and magnetic resonance imaging (MRI) (which is better than CT) are the best means of analysis of the cyst [7, 8]. In the present case, CT was used because MRI was too expensive for our patient. The majority of the cysts are asymptomatic and regress spontaneously [9]. When the cyst is large, it can compress the gravid uterus, slow the fetus intra-uterine growth, cause premature delivery or abnormal presentation of the fetus. In the postpartum, the giant cyst is a risk factor for haemorrage. In our case, vaginal delivery has been possible with no complications.

The differential diagnosis of an abdominal mass includes benign and malignant gynecologic and non-gynecologic etiologies. A giant ovarian cyst can provoke a differential diagnostic problem with another fluid abdominal mass. In the present case, imaging had evoked both a giant ovarian cyst and a huge mesenteric cyst. Correct preoperative diagnosis is quite difficult due to the rare occurrence or the lack of specific clinical presentation of the giant ovarian cyst. Common symptoms, due to compressive effect such as abdominal pain, distension, bloating, constipation and vomiting can arise. Before surgery, two main arguments made us to think of a giant ovarian cyst rather than a huge mesenteric cyst: the female sex of our patient, and the rarity of mesenteric cysts. In fact, mesenteric cysts are often found among the paediatric population with an annual incidence of 1 for 20,000 and are very rare in the adult population with an annual incidence of only 1 for 100,000 [10].

The management approach depends on the size of cyst, equipment, and level of surgeon’s experience. According to many authors, aspiration of the contents of the cyst should be avoided because of complications such as infection, bleeding, rupture of the cyst, increased risk of peritoneal adhesion [11–13]. Yet, surgery can be done laparoscopically. In so doing, after the introduction of the trocars, an aspiration of the contents of the cyst is made before its removal [14]. But this laparoscopy is not recommended when the cyst is suspected of malignancy because of the risk of spreading cancer cells [13, 14]. In our case, we preferred a laparotomy because we were not sure of the benign nature of the cyst preoperatively. Furthermore, laparoscopy surgery was not possible due to the large size of the cyst.

An immediate complication to be feared when removing a giant ovarian cyst is the vacuum shock requiring a good preventive vascular filling [15]. In the literature, cases of giant ovarian cysts during pregnancy or postpartum have rarely been reported. Qublan et al. [16] in 2002 removed a 6 kg ovarian mucinous cyst after caesarean section. Petros et al. [17] removed a 30 × 25 mm bilateral mucinous benign ovarian cyst in 2005. As for Noreen et al. [18], in 2011, they reported a giant ovarian cyst discovered at 32 weeks of gestation and which was removed at 38 weeks of gestation through an oophorectomy. In 2017 Baradwan et al. [19] removed a 16.5 × 26.3 × 22.4 cm ovarian serous cystadenoma laparoscopically in the postpartum. All these cysts in the gravid-puerperium period did not have the size of our cyst, which measured 42 cm long-axis and weighed 19.7 kg. Except its large size, the cyst in our patient had no other malignancy. Worth noting is that, ultrasound features that increase the suspicion of malignancy are loss of any normal ovarian tissue surrounding the cyst and the existence of solid areas or papillary projections within the cyst. However, borderline tumors can be difficult to differentiate from benign tumors on the basis of ultrasound image characteristics.

Most cysts in the gravid-puerperium period are functional and therefore benign. It is often a luteoma of pregnancy. The other ovarian cysts encountered during pregnancy are, in order of frequency, benign teratomas, mucosal adenomas, rete ovarii tumors and endometriotic cysts [20]. A serous cystadenoma is a commonest benign ovarian cyst and accounts for approximately 60–75% of ovarian cysts. They are the benign epithelial tumors and are usually unilateral and uni-locular. Their incidence tends to peak at 20–40 years. But the aetiology of serous cysts is unknow, although they may be associated with other ovarian tumors such as mature cystic teratomas. Cheng et al. [21] demonstrated that mutations in BRAF and KRAS that characterize serous borderline tumors and low-grade serous carcinomas are absent in serous cystadenomas. They speculated that a small proportion of these cystadenomas become clonal and that mutations of KRAS or BRAF in some of these clonal cystadenomas lead to the development of serous borderline tumors, which are the precursors of low-grad serous carcinoma.

After surgery, because our patient’s cyst was benign, the long-term risks were supposed to be very reduced. Those risks are related to surgery rather than pathology. Indeed, it can be argued that the occurrence of adhesions of intra-abdominal organs is possible after surgery. Also, the ovariectomy performed may slightly reduce fertility and decrease the age of onset of menopause.

This case report proves that vaginal delivery is possible in the association of giant ovarian cyst and pregnancy. Surgical management of a giant cyst was performed in the postpartum with satisfaction. This cyst, histologically, was known as benign. Indeed, for early diagnosis, a better evaluation through both clinical and systematic ultrasound, during antenatal period and intrapartum, should be encouraged even in low-resource countries.

## Data Availability

Data are available Yalgado Ouedraogo University Hospital Center archives and can be sent by corresponding author on request.

## Abbreviations

CA-125: Carbohydrate antigen 125
CT: Computed tomography
MRI: Magnetic resonance imaging
